# Supplementary material for: Determinants of multidrug-resistant urinary tract infections: A retrospective cross-sectional study from a tertiary care hospital in southern Bangladesh
Source: PLOS Glob Public Health. 2026 Jan 16;6(1):e0005022. doi: 10.1371/journal.pgph.0005022 (PMC12810910; doi:10.1371/journal.pgph.0005022)
Supplement: Attachment — Submitted filename: Rebuttal Letter_14 Nov.docx [file pgph.0005022.s001.docx]

**Rebuttal Letter**

**Dear Editor,**

Thank you for your detailed feedback and thoughtful comments on our manuscript titled "Determinants of Multidrug-Resistant Urinary Tract Infections: A Retrospective Cross-Sectional Study from a Tertiary Care Hospital in Southern Bangladesh" (PGPH-D-25-02034). We truly appreciate the time and effort you and the reviewers put into evaluating our work.

We have carefully addressed all the points raised and revised the manuscript accordingly. Below, we provide a detailed, point-by-point response to the comments made by the reviewers.

| **Reviewers’ Comments** | **Authors’ Responses** |
| --- | --- |
| Potential Copyright Issues:  Figure 1:  please (a) provide a direct link to the base layer of the map (i.e., the country or region border shape) and ensure this is also included in the figure legend; and (b) provide a link to the terms of use / license information for the base layer image or shapefile. We cannot publish proprietary or copyrighted maps (e.g. Google Maps, Mapquest) and the terms of use for your map base layer must be compatible with our CC-BY 4.0 license. | We confirm that the base layer for Figure 1 utilizes a publicly available shapefile whose usage terms are compatible with the journal's Creative Commons Attribution 4.0 International (CC BY 4.0) license.  We have addressed the reviewer's points as follows:  **Direct Link to the Base Layer (and inclusion in the Figure Legend):**   - The shapefile for the map base layer was sourced from DIVA-GIS (<https://diva-gis.org/data.html>). This link has been included in the updated figure legend   Fig 1. Geographical Distribution of obtained Urine Samples... The map base layer was generated using a public domain shapefile from DIVA-GIS (<https://diva-gis.org/data.html>). |
| 1. Inconsistencies in numbers The abstract mentions 1,797 urine samples, whereas the main text reports 1,697. Please check and harmonize these numbers. | We have reviewed the manuscript and would like to clarify that the correct total number of urine samples is 1,797, as mentioned in lines 128-132 of the manuscript. The breakdown of these samples is as follows: 1,420 samples from the outpatient department (OPD), 180 from specialized units (SUs), 70 from private medical settings (PMSs), and 127 samples with unspecified sources. We didn’t proceed the analysis with data with unspecified sources because they lacked proper information. There is no mention of 1,697 samples in the manuscript. . We have ensured consistency throughout the text, and the number of 1,797 is accurate and reflects the total number of samples correctly. No changes are needed in this regard. |
| **2. Study population and inclusion criteria:**  a) Inclusion criteria are not clearly described (hospitalized patients? children included? sampling method?).  b) The presence of patients from surgery/urology suggests specific risk factors (surgery, urinary catheters) that should be clarified and discussed. | A) We have clarified the inclusion criteria and the study design and duration in the revised manuscript (Line: 122-138). This is retrospective cross-sectional study was conducted using an **anonymized secondary dataset** comprising urine culture and sensitivity test results collected between January and December 2023 (Line: 123-125). The present study did not involve direct contact with patients or any additional sample collection beyond routine clinical procedures (Line: 134-136)  b) Discussed |
| **3. Microbiological methods:**  a) Questionable use of Gram staining on MacConkey agar.  b) Gram-positive bacteria: refer to presumptive rather than definitive identification.  c) Lack of standardized inocula may bias susceptibility testing.  d) Reported incubation time (16–18 h) is shorter than CLSI standards: please verify and reference.  e) Antibiotics should be written in lowercase and grouped by class.  f) Clearly distinguish quinolones from fluoroquinolones. | a) Gram staining was not performed directly on MacConkey agar. In our study, Gram staining was performed on bacterial isolates after they had been cultured on Sheep Blood Agar and MacConkey Agar, both of which are standard media for isolating and identifying uropathogens.  b) We agree that the term "presumptive" is often used when molecular methods are not employed, we would like to clarify that the biochemical tests we used for Gram-positive bacteria identification, specifically catalase and coagulase tests, are standard and well-established methods for confirming bacterial species within the *Staphylococcus* genus. These tests allowed for a reliable identification of *Staphylococcus* spp., distinguishing them from other Gram-positive organisms. Given that these biochemical methods are widely accepted in clinical microbiology for routine identification and have been used effectively in our study, we believe the identification of *Staphylococcus* spp. in this context is definitive, though we highly acknowledge that molecular methods could provide additional confirmation. Due to lack of funds, molecular assessments couldn’t be done.  (<https://ann-clinmicrob.biomedcentral.com/articles/10.1186/1476-0711-9-23>)    c) we ensured that bacterial suspensions were adjusted to match the 0.5 McFarland standard to maintain consistent inoculum density for each test. While minor variations may still occur, this approach is widely used and considered adequate for routine AST in clinical microbiology.  D) The pathogens isolated in our study are generally considered non-fastidious organisms. They grow well on standard laboratory media such as MacConkey agar and Sheep Blood agar without the need for enriched or specialized growth conditions.  e) Regarding the incubation time for AST, our study adhered to the CLSI M100 guidelines, which recommend an incubation period of 16–18 hours at 35°C in ambient air for disk diffusion testing. This duration is considered optimal for the growth of most aerobic bacteria and the accurate determination of AST.  f) done (Line: 187-197) |
| **4. Definition of multidrug resistance:**  MDR definition is missing. Please provide the exact definition with an appropriate reference (e.g., Magiorakos et al., 2012, CMI). | Its already added in the introduction section (Line 58-59). We replaced the paper in no 1 ref. |
| **5. Presentation of results:**  a) Table 1: Only four species isolated from 229 positive urines seems surprising → clarify.  b) Add a table showing susceptibility profiles used to define MDR cases.  c) Table 2: presentation unclear → specify whether it compares MDR+ vs MDR− or between categories.  d) MCA: mentioned but not shown → add a figure. | a) The reason for isolating only four species from the 229 positive urine samples is due to the specific selection criteria employed in our study. We focused on the most common and clinically relevant uropathogens that are typically responsible for UTIs in southern Bangladesh, particularly in the context of MDR. The four species identified are well-known and frequently encountered pathogens in UTIs and are highly relevant to the study of AMR  b) Pls check **figure 3**  c)Table 2 presents the univariate analysis comparing MDR-positive (MDR+) cases versus MDR-negative (MDR−) cases across different categories. The table includes a comparison of factors such as age, sex, sample source, and bacterial species to assess their association with the presence of MDR. The goal of this analysis was to identify which variables are significantly associated with MDR status.  d) Added both the figure and the plotting graph! Pls check **figure 3** |
| **6. Interpretation of results:**  a) Lines 341–347: significant association between MDR and male sex is unexpected → provide possible explanations (e.g., catheters, prostatic disease).  b) Line 367: low prevalence in children should be explained (immature immunity, reflux, lack of circumcision, malnutrition). | a) It was already given. Pls check Line: 389-393  b) Done (Line: 425-434) |
| **Minor Comments:**  a) Abstract: report MDR prevalence (60/229 = 26.2%).  b) Abstract and text: simplify overly long sentences.  c) References: provide those for culture media used.  d) Abstract: rephrase 'highlighting primary and secondary contributors' for clarity.  e) Antibiotics: harmonize formatting (lowercase, grouped by classes).  f) Text: shorten some sentences for readability. | a) It was already written. The PR will be 30.56% not 26.2% (Line: 37-38)  b) Done  c) Done    d) Done  e) harmonized and keep it in lowercases  f) shorten some sentences in abstract and introduction . |

We believe that the revisions have significantly improved the clarity and rigor of our manuscript. We thank the reviewers again for their insightful comments.

Sincerely,
**Dr. Abu Sayed DVM (PSTU, Bangladesh); CLT (CAAS, China)**
Erasmus Mundus Scholar

MSc Candidate in International Vaccinology (LIVE)

Université Claude Bernard Lyon 1(UCBL), Lyon, France

Universitat Autònoma de Barcelona (UAB), Barcelona, Spain
Universitat de Barcelona (UB) Barcelona, Spain
Antwerpen Universiteit (UA) Antwerp, Belgium

Université Jean Monet (UJM), Saint Étienne, France

**Email:**

[a.sayedpstu@gmail.com](mailto:a.sayedpstu@gmail.com)

**Phone:** (+34) 635980015
